# Fibroblasts in heterotopic ossification: mechanisms and therapeutic targets

**DOI:** 10.7150/ijbs.102297

**Published:** 2025-01-01

**Authors:** Jia-xin Li, Yan-miao Dang, Meng-chao Liu, Lin-qing Gao, Hui Lin

**Affiliations:** 1 School of Basic Medical Sciences, Jiangxi Medical College, Nanchang University, Nanchang, 330006, China.; 2 First Clinical School, Jiangxi Medical College, Nanchang University, Nanchang, 330006, China.

**Keywords:** Heterotopic ossification, Fibroblast heterogeneity, Therapy, Cell cross-talk

## Abstract

Heterotopic ossification (HO) refers to the abnormal formation of bone in non-skeletal tissues. Fibroblasts have traditionally been viewed as stationary cells primarily responsible for producing extracellular matrix during tissue repair and fibrosis. However, recent discoveries regarding their plasticity—encompassing roles in inflammation, extracellular matrix remodeling, and osteogenesis—highlight their potential as key contributors to the development of HO. In this review, we systematically summarize the diverse phenotypic and functional plasticity of fibroblasts in HO. Furthermore, we evaluate the possible interaction between fibroblasts and macrophages in pathophysiological processes and signaling pathways. Finally, we highlight the potential strategies for preventing and treating HO by targeting fibroblast activities.

## Introduction

Heterotopic ossification (HO) refers to the pathological formation of bone in non-skeletal tissues, such as muscles, joints, and blood vessels. It is often considered a result of defective tissue repair and commonly occurs as a complication of trauma, burns, surgery, or some systemic injuries^1^. HO is classified into genetic and acquired forms, both of which share a common pathological mechanism involving the reactivation of the bone formation program triggered by inflammation^2^. The development of heterotopic bone requires three key elements: osteogenic progenitor cells, molecular signals that initiate bone formation, and a conducive environment that supports bone formation^3^. On this basis, HO undergoes four sequential processes: injury/inflammation, stem cell recruitment, chondrogenic differentiation, and finally ossification. The primary mechanisms of osteogenesis are intramembranous ossification and endochondral ossification, with the latter being more prevalent in clinical cases, which usually occurs in neurogenic, traumatic, and degenerative HO^4^. HO significantly impairs patients' quality of life by causing discomfort and restricting movement. Despite its impact, the underlying molecular mechanisms remain largely unknown, and the current therapeutic approaches, which encompass nonsteroidal anti-inflammatory drugs (NSAIDs) and surgical interventions, provide only restricted alleviation^5^. Identifying the molecular and cellular drivers of HO is crucial for developing effective therapeutic strategies.

Single-cell RNA sequencing has revealed that fibroblasts are the key cell population in HO^6^. Several studies have demonstrated that fibroblasts may play a pivotal role in all stages of the HO process. They mediate inflammation to initiate bone formation, create a conducive microenvironment characterized by fibrosis, hypoxia, and extracellular matrix (ECM) remodeling, and serve as osteogenic progenitor cells^7^-^9^. Fibroblasts initiate bone formation by recruiting leukocytes and promoting an inflammatory infiltrate. In this process, they interact with immune cells, transforming acute inflammation into chronic inflammation, which can lead to fibrosis and defective tissue repair^10^. At the same time, as major ECM producers, they can create an environment characterized by low oxygen levels, angiogenesis/vascular permeability, stromal remodeling, and metabolic reprogramming. This environment promotes the formation of heterotopic bone and serves as a supporting structure for cells capable of differentiating into bone tissue^11^. Notably, fibroblasts have a strong capacity for osteogenesis and can function as progenitor cells directly involved in bone formation in ectopic sites^12^.

This review discusses the various roles of fibroblasts, a key cellular component among the three essential factors in the development of HO, as well as the signaling pathways and mechanisms involved in this process. Furthermore, we elucidate the potential interactions between fibroblasts and macrophages in different types of HO (FOP and traumatic HO). These complex interactions may offer new insights into the persistent, recurrent, and refractory nature of HO and provide a research direction for muscle injury-related diseases. Finally, based on the topics discussed above, we outline potential therapeutic strategies targeting fibroblasts across various pathological processes of HO, including inflammation, fibrosis, ECM remodeling, and differentiation of ectopic bone precursor cells. These strategies include technologies such as nanomedicine and photodynamic therapy. We hope to provide distinctive insights for the prevention, early detection, and treatment of HO.

## Mechanisms in different types of heterotopic ossification

### Genetic heterotopic ossification

Genetic heterotopic ossification comprises three types: progressive osseous heteroplasia (POH), Albright hereditary osteodystrophy (AHO), and fibrodysplasia ossificans progressiva (FOP)^13^. POH represents the most severe form of HO, typically manifesting in infancy and leading to early mortality due to extensive extraosseous bone formation, even following minor trauma^14^. AHO is a complex disorder marked by developmental abnormalities, frequently associated with parathyroid hormone (PTH) resistance^15^. Patients diagnosed with POH and AHO typically have mutations in the GNAS gene, leading to a loss or reduction in G protein alpha subunit (Gsα) function. This disrupts the adenylate cyclase system, causing abnormal cAMP signaling and, consequently, uncontrolled bone formation^13^.

FOP is the most common form of genetic HO, characterized as a severely disabling inherited disease with a global prevalence of 1/1,300,000-1/2,000,000. This condition does not exhibit racial, ethnic, or regional predisposition^16^. Clinical manifestations include progressive heterotopic ossification, congenital first toe deformity, and accelerated aging^17^. In the early stages of FOP, patients may experience intermittent inflammatory soft tissue swellings, referred to as flare-ups. When exposed to factors such as injury, intramuscular injections, viral infections, muscle overuse, or fatigue, these flare-ups will ultimately lead to the transformation of connective tissues into ectopic bone^18^.

**Figure 1:**
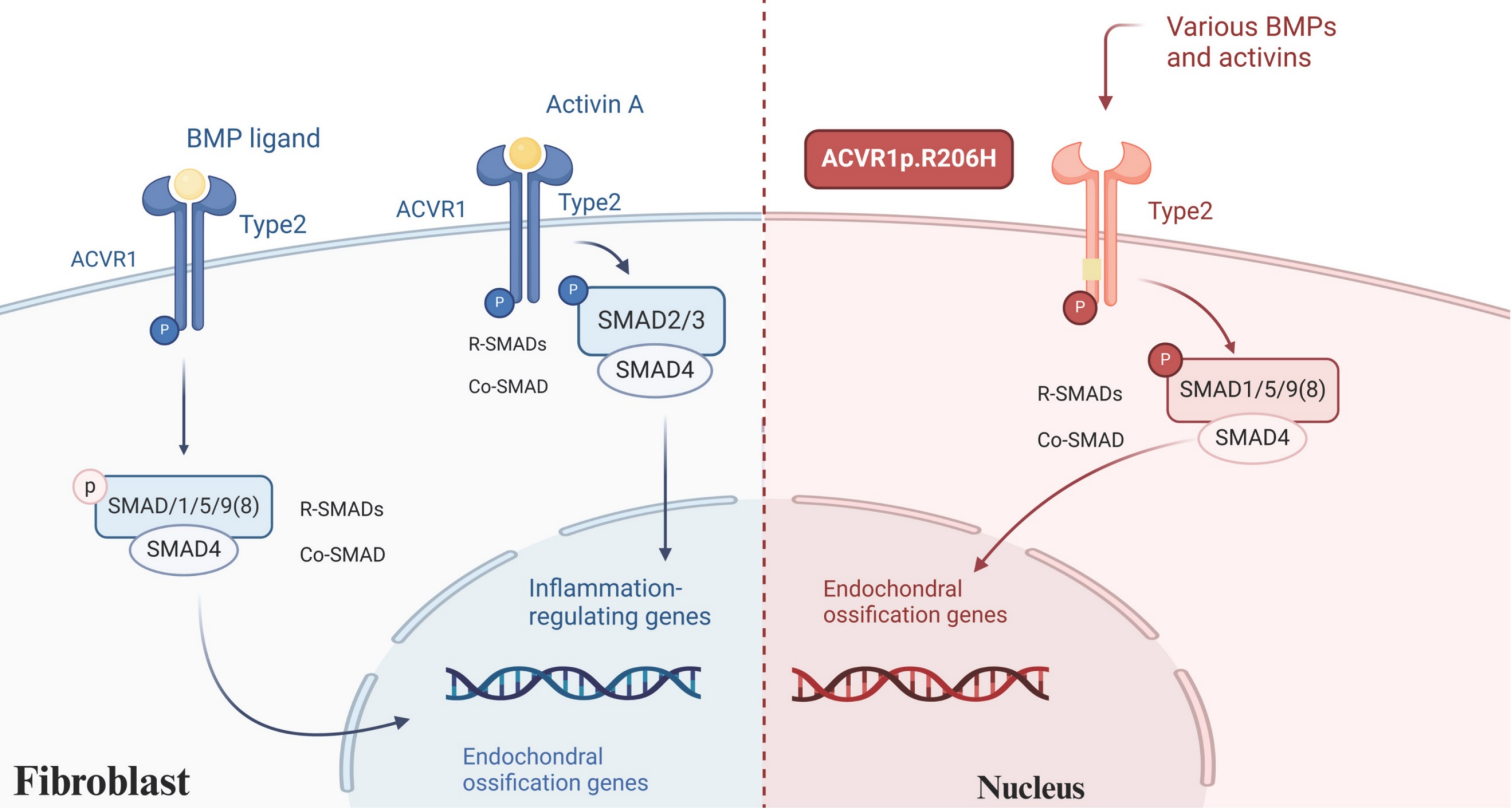
** BMP signaling in FOP.** BMP activates wild-type ACVR1, triggering an osteogenic response through SMAD 1/5/8 signaling. In contrast, Activin A inhibits BMP signaling in wild-type ACVR1 and functions within the TGF-β pathway. However, in mutant ACVR1 Activin A aberrantly activates SMAD 1/5/8 signaling, promoting the differentiation of MSCs into osteoblasts. (Created in BioRender.com).

Mutations in the single nucleotide of the ACVR1 gene (also referred to as ALK2) are identified in approximately 97% of FOP patients, particularly the c.617G>A mutation, which results in an arginine-to-histidine substitution at codon 206 (p.R206H)^16^, ^19^. This mutation induces a conformational change in the intracellular GS structural domain of the receptor, enhancing its sensitivity and activity, and consequently hyperactivating the downstream bone morphogenetic protein (BMP) signaling pathway^20^. Currently, it is recognized that the BMP signaling pathway holds a pivotal role in the pathophysiology associated with HO. BMPs possess the capability to elicit bone formation in skeletal muscle within living organisms and stimulate the differentiation of progenitor cells towards osteoblasts in controlled environments^21^. In addition to the aberrant activation of ACVR1, emerging evidence highlights the significant role of Activin A, a member of the transforming growth factor-beta (TGF-β) superfamily, in FOP. Activin A, produced by immune cells, plays a marked role in mediating fibrosis, inflammation, and immunity^22^, ^23^. Normally, BMP activates wild-type ACVR1 and then utilizes SMAD 1/5/8 signaling to trigger an osteogenic response. Activin A participates in TGF-β signaling and acts as a competitive inhibitor of wild-type ACVR1/BMP signaling^24^. However, when ACVR1 is mutated, it can respond to Activin A by activating similar SMAD 1/5/8 and mTOR signaling pathways, leading to the differentiation of mesenchymal stem cells (MSCs) into osteoblasts while inhibiting the formation of osteoclasts^25^** (Figure 1)**. The application of Activin A antibodies in a mouse model of FOP effectively halted the progression of HO, underscoring the potential of neutralizing antibodies as a promising therapeutic approach for FOP^26^. Although mutations in ACVR1 do not directly cause severe pro-inflammation responses in FOP, levels of Activin A in the bloodstream increase significantly following inflammatory invasion episodes^27^. Consequently, when the abnormal activity of the mutated receptor is triggered, even mild inflammation and the absence of prior trauma can provoke soft tissue swelling or flare-ups^28^. Furthermore, FOP exhibits similarities with other forms of HO, incorporating the participation of hypoxia and angiogenesis-related signaling pathways^29^.

### Acquired heterotopic ossification

**Figure 2:**
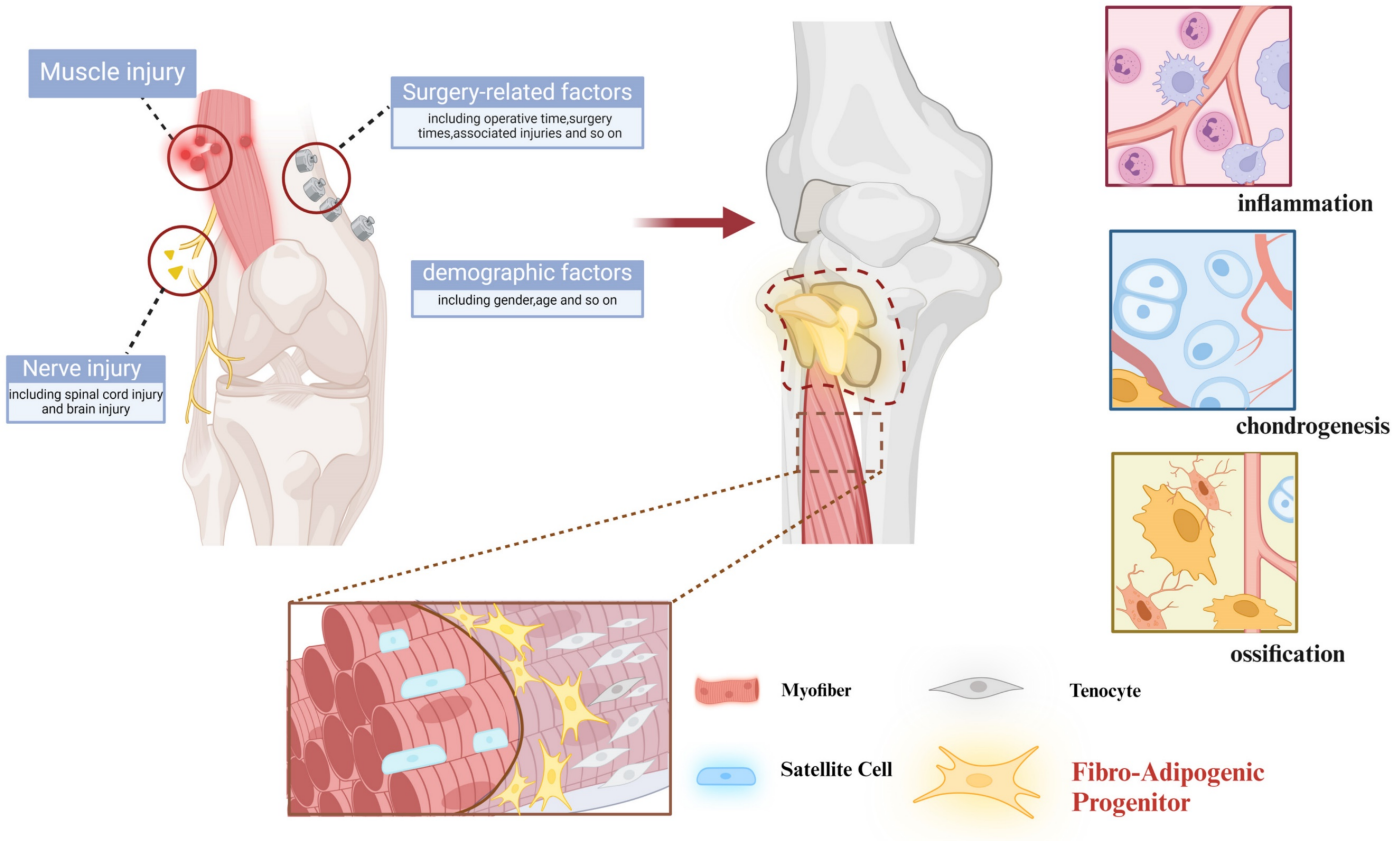
** Mechanisms in acquired heterotopic ossification.** Acquired heterotopic ossification generally occurs after severe trauma, burns, surgery, or central nervous system injury and induces inflammation and hypermetabolism, which ultimately triggers the activation of stem cells with heterotopic endochondral ossification to form heterotopic bone. (Created in BioRender.com)

Unlike genetic heterotopic ossification caused by mutations, acquired heterotopic ossification typically develops following severe trauma, burns, surgery, or central nervous system injury^30^-^32^. It results from improper wound healing, systemic or local immune activation, and infections, which lead to inflammation and hypermetabolism. This, in turn, stimulates stem cells to ossify within ectopic cartilage, eventually forming ectopic bone^33^, ^34^. Post-traumatic HO progresses through a highly regulated process known as endochondral ossification, wherein cartilage intermediates are replaced by bone. During this process, endothelial cells invade the site, facilitating new blood vessel formation, while osteoblasts are recruited to produce the bone matrix^3^ **(Figure 2)**.

The prevalence of acquired HO is substantial affecting over 30% of patients following orthopedic procedures such as hip replacement. In individuals with war-related injuries, the incidence can exceed 60%, and in cases of severe traumatic amputations, it may surpass 90%^35^-^37^. Common symptoms of HO include chronic pain, restricted joint mobility, and impaired movement. Nowadays, the treatments for acquired HO are limited to radiation therapy, NSAIDs, and surgery, which have significant recurrence rates^38^, ^39^

## Mechanisms implicated in fibroblasts in HO

Fibroblasts were originally identified as spindle-shaped connective tissue cells enveloped by their collagen fibers, and at that time were considered a uniform cell population^40^. However, recent developments in cell lineage tracing and single-cell transcriptomics have revealed that fibroblasts are a heterogeneous and partially overlapping group^41^.

Fibroblasts arise from various sources depending on the specific tissue and physiological context. They can be derived from MSCs, which differentiate into fibroblasts during tissue development, repair, and maintenance. In certain situations, such as wound healing or fibrosis, epithelial cells may undergo epithelial-mesenchymal transition (EMT) and transform into fibroblasts. Furthermore, pericytes—the cells that envelop blood vessels—have been demonstrated to differentiate into fibroblasts in response to injury^42^.

Although fibroblasts are differentiated cells of the mesenchymal lineage, derived from MSCs, research suggests that under specific conditions, they can acquire osteogenic-like properties through bone-related signaling pathways, functioning similarly to MSCs. A study utilizing single-cell RNA sequencing to investigate fibroblast heterogeneity in keloids identified a subpopulation of mesenchymal fibroblasts. This subpopulation expressed mesenchymal progenitor markers, such as COL11A1 and POSTN, which play roles in skeletal system development, osteogenesis, and osteoblast differentiation. These findings highlight the plasticity of fibroblasts and their potential role in processes traditionally attributed to MSCs^41^.

However, the various subtypes of fibroblasts have not yet been fully elucidated. Buechler *et al.* utilized single-cell RNA sequencing to analyze the transcriptomes of approximately 230,000 fibroblasts, constructing a comprehensive fibroblast atlas. The study identified two ubiquitous transcriptional subtypes (Pi16+ and Col15a1+) across different tissues. These subtypes serve as reservoirs capable of differentiating into tissue-specific fibroblasts under homeostatic conditions and into activated fibroblasts during disease^43^, ^44^.

Additional specialized fibroblast subtypes were also discovered, such as the CCL19+ subtype associated with fibroblastic reticular cells (FRCs) in lymph nodes, the CXCL12+ subtype linked to MSCs and osteoblasts, and the Comp+ subtype related to chondrocytes^44^.

Furthermore, the study revealed that under disease or tissue injury, fibroblasts can further differentiate into specific activated states, such as cancer-associated fibroblasts (CAFs) in tumors or myofibroblasts in fibrotic diseases. These activated fibroblast subtypes are typically involved in inflammation, tissue repair, and pathological processes^45^.

### Heterogeneity of fibroblasts in HO

As previously mentioned, fibroblasts exhibit diverse functions, yet their specific classification has not been fully elucidated. Researchers have categorized fibroblasts into four layers, suggesting that each fibroblast's transcriptomic profile and phenotype are shaped by these four layers: tissue condition, anatomical heterogeneity, local microenvironment (including extracellular matrix, cell-stroma interactions, paracrine and autocrine signaling), and cellular phenotype (such as quiescent, proliferative, senescent, activated, migratory or differentiated state)^46^. Fibroblasts exhibit distinct functional properties depending on the layer they reside in.

**Figure 3:**
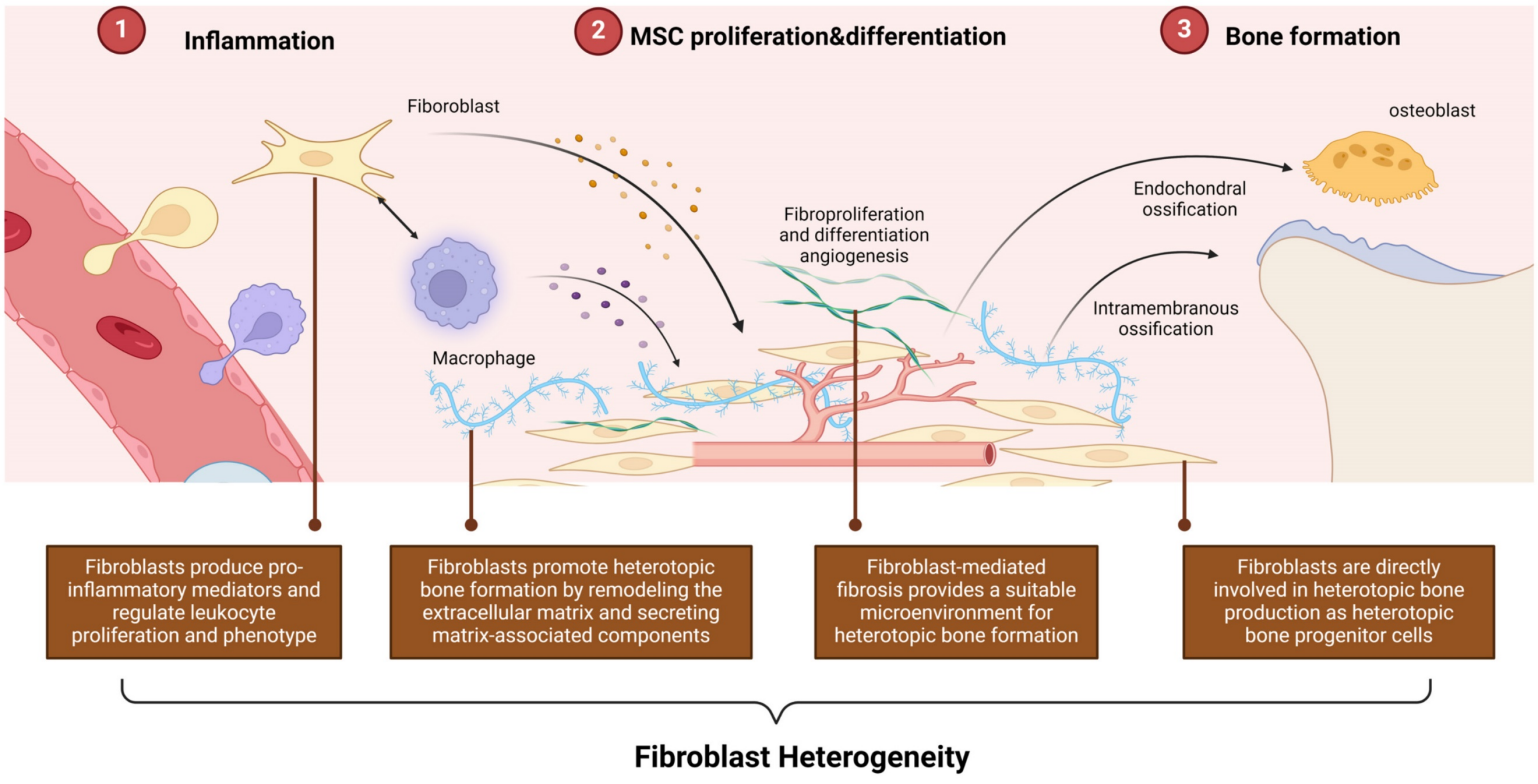
** Fibroblast Heterogeneity in HO.** Based on the heterogeneity, fibroblasts can be involved in all stages of the HO process by acting as mediators between inflammation, tissue repair, and osteogenic differentiation. (Created in BioRender.com).

Given the heterogeneity of fibroblasts, understanding the specific roles of these layers in HO is crucial and warrants further clarification **(Figure 3)**.

#### Fibroblast aging

Aging is marked by systemic chronic inflammation linked to cellular senescence, immunosenescence, organ failure, and age-related illnesses^47^. Senescent cells release a variety of molecules known as senescence-associated secretory phenotype (SASP), which include cytokines, chemokines, and matrix proteases such as IL-6, COL1A1, CCL2, and Activin A^48^.

Certain SASP factors have been shown to induce osteogenesis, particularly in the context of muscle trauma, where they promote tissue remodeling and cellular reprogramming, converting neighboring cells into osteogenic progenitors in traumatic HO^49^, ^50^.

Mahmoudi *et al.* utilized multi-omics analyses, including transcriptomics, epigenomics, and metabolomics, to assess fibroblast heterogeneity in young and aged mice. The study revealed that fibroblasts from aged mice released more inflammatory cytokines and showed more diversity in reprogramming effectiveness among individuals. Moreover, aged fibroblasts included a larger fraction of “activated fibroblasts,” characterized by cytokine secretion, with this proportion being closely linked to reprogramming efficiency^51^. Fibro/adipogenic progenitors (FAPs), which are highly susceptible to senescence, play a crucial role in muscle regeneration by remodeling the ECM^52^. In older animals, senescent FAPs exhibit a more pro-inflammatory transcriptional profile^53^.

As a result, fibroblast senescence may contribute to abnormal ECM remodeling, impair muscle regeneration, and ultimately promote the development of HO.

The researchers analyzed scRNA-seq data from HO samples and identified a potential link between injury-related senescent fibroblasts and HO. These fibroblasts likely mediate their effects through PI3K/Akt/mTOR-induced SASPs^4^, which may promote the conversion of neighboring cells into osteogenic progenitor via SASP secretion^54^. Suppression of nuclear factor kappa-light-chain-enhancer of activated B cells (NF-κB), a key factor in SASP formation, allows fibroblasts to evade Akt-induced senescence and reduce the likelihood of *in vivo* tissue reprogramming. Moreover, treatment of senescent cells with rapamycin, a mTORC1 inhibitor that acts downstream of PI3K/Akt, resulted in a 35% reduction in SASP secretion and NF-κB transcription^4^, ^55^.

While the link between senescent fibroblasts and HO is becoming clearer, significant limitations remain. Further molecular, cellular, and animal studies are needed to fully elucidate the underlying mechanisms and signaling pathways. Furthermore, more research is needed to identify the specific cell types targeted by SASPs released from senescent fibroblasts that are eventually reprogrammed into progenitor cells involved in HO.

#### Other heterogeneity of fibroblasts

In addition to the heterogeneity determined by the cellular phenotype, different subsets of fibroblasts may play distinct roles in HO, contributing to processes such as inflammation, ECM production, fibrosis, and osteogenesis. Each of these functions will be discussed in detail below.

### Fibroblast-mediated inflammation triggers osteogenesis in HO

Inflammation is recognized as a critical trigger for both FOP and traumatic HO^56^, ^57^. Historically considered “immune-neutral cells”, fibroblasts were once supposed to play a passive role in inflammation^58^. However, recent studies have revealed that fibroblasts can function as immunological sentinels. They detect damage-associated molecular patterns (DAMPS) and pathogenesis-associated molecular patterns (PAMPS), activating pro-inflammatory signaling pathways and releasing pro-inflammatory mediators such as IL-6, PGE2, GM-CSF, and CCL2. These mediators attract immune cells to the site of inflammation^59^, ^60^. Moreover, studies show that interactions between leukocytes and fibroblasts enhance leukocyte survival, development, and specialization influencing their functional properties^61^. These inflammatory processes can ultimately activate osteogenic signaling in HO precursor cells.

Researchers studied the role of FAPα+ fibroblasts *in vivo* by selectively depleting them during arthritis. Deleting FAPα+ cells decreased leukocyte infiltration and was inversely associated with joint inflammation severity^62^. Removing FAPα+ fibroblasts reduced the expression of osteoclast and osteoblast markers in joint tissue^62^.

Ossification of the posterior longitudinal ligament (OPLL) is a progressive condition marked by abnormal bone formation in the spinal ligaments^63^. Connexin 43 (Cx43), a gap junction protein highly present in bone, was discovered to be considerably increased in OPLL fibroblasts in response to mechanical loading. Ligament fibroblasts treated with si-Cx43 exhibited a marked reduction in phosphorylated p65 protein levels in both OPLL and non-OPLL cells under mechanical stress^64^. OPLL fibroblasts treated with si-Cx43 and an NF-кB (p65) pathway inhibitor produced much lower levels of inflammatory proteins such as IL-1α, IL-6, and TGF-β. The expression of osteoblast markers ALP, COLI, and CON was also downregulated. The results indicate that Cx43 is crucial in triggering the inflammatory response in OPLL fibroblasts through activation of the NF-кB (p65) pathway, which subsequently enhances their osteogenic differentiation^64^.

Throughout the progression of HO, fibroblasts trigger inflammatory reactions, which can be mitigated through the activation of the adenosine 5'-monophosphate-activated protein kinase (AMPK) signaling cascade^65^.

Ankylosing spondylitis (AS) is a chronic inflammatory disease with a strong genetic predisposition, primarily affecting the spine and pelvic region. A key characteristic of AS is endosteal HO, triggered by post-inflammatory mechanisms, which ultimately leads to joint ankylosis^66^, ^67^. In AS, the expression of PI3k, Akt, and AMPK mRNA is reduced. However, activation of AMPK inhibits inflammatory factors like TNF-α, IL-1β, and IL-6, while upregulating PI3K and Akt expression in fibroblasts^68^. Additionally, activation of the PI3k/Akt pathway has been demonstrated to attenuate inflammation^69^. Thus, AS may increase inflammatory factor expression by downregulating this pathway, while AMPK activation can suppress inflammation and potentially prevent HO.

### Fibroblast-mediated extracellular matrix remodeling promotes osteogenesis in HO

HO is commonly initiated by tissue injury, disrupting normal homeostasis and altering the composition and structure of the ECM.

Fibroblasts, as the primary producers of ECM components, play a central role in ECM remodeling^70^. The ECM itself significantly impacts cellular function and phenotype, influencing osteoblasts, osteoclasts, and fibroblasts alike^71^. Fernandes *et al.* found that fibroblast-secreted growth factors significantly upregulated the expression of osteoblast-specific gene markers, such as dentin matrix protein 1 (DMP1) and sclerostin (SOST), while also promoting the expression of matrix metallopeptidase 9 (MMP-9) and reversion-inducing cysteine-rich protein with kazal motifs (RECK), which are associated with ECM remodeling. Additionally, the study demonstrated that fibroblasts had a notable impact on the expression of inflammatory factors such as IL-13 and IL-33, both of which are closely related to osteoblast differentiation and function^72^.

Moreover, the mechanical properties of the ECM, such as tension, stiffness, elasticity, strain, and adhesion, can also affect MSCs differentiation and contribute to HO development^73^.

#### Collagen in ECM promotes bone deposition

HO is an active metabolic process where fibroblasts secrete matrix components that facilitate the formation of ectopic bone deposits. HO can also be categorized as a proliferative disorder marked by the abnormal accumulation of bone and cartilage matrix within non-skeletal tissues.

Immunohistochemical analysis of tissues from paraplegic patients with pressure ulcer lesions revealed that Proteoglycan-100 expression is associated with specific stages of HO. Notably, in the preosteogenic phase, Proteoglycan-100 is predominantly expressed in fibroblasts and osteogenic progenitor cells^74^.

Fibroblasts secrete various types of collagen that contribute to the abnormal growth of bone tissue^75^. Spinal stenosis partially arise from hypertrophy and ossification of the ligamentum flavum (LF), which are processes fueled by degenerative alterations in ligament fibroblasts, encompassing heightened collagen synthesis and chondroid metaplasia. Researchers have found that in cases of spinal stenosis, the release of inflammatory mediators such as IL-6, TNF-α, PGE2, and NO stimulates fibroblasts to produce type III, V, and XI collagen, which facilitates the formation of bone nodules within the ligament^76^.

Medina *et al.* used flow cytometry to isolate HO cells, identifying that a substantial proportion (up to 65%) displayed dual staining for lymphocyte-specific protein 1 (LSP1) and type I collagen during cell expansion. Immunofluorescence microscopy further revealed these cells' spindle-shaped, fibroblastic morphology^77^. In addition, these fibroblast-like cells contribute to the formation of a nascent organic matrix, which has yet to mineralize. Elevated hydroxyproline levels in these cells indicate that they produce significantly more collagen compared to regular fibroblasts. The non-mineralized matrix plays a pivotal role in new bone formation and is a critical component of HO lesions^77^.

Biphasic calcium phosphate (BCP) ceramics have been shown to stimulate heterotopic bone formation when implanted subcutaneously or intramuscularly in animals^78^. Additionally, certain studies have developed bioactive hydrogel scaffolds designed to modulate fibroblast migration, collagen synthesis, and cytokine secretion effectively modifying the extracellular matrix environment^79^.

Fu *et al.* developed bioactive BCP ceramics, which were used in an ectopic bone formation model to induce osteogenesis. HE staining showed a significant accumulation of fibroblasts around the ceramic seven days after implantation. qRT-PCR results indicated an increase in Col1a1 expression. Additionally, ALP staining further showed that BCP ceramics facilitated the osteogenic differentiation of BMSCs via fibroblasts. Interestingly, osteogenic factors such as BMP2 and insulin-like growth factor 1 (IGF1) were markedly upregulated at the mRNA level in BCP-treated L929 fibroblasts, suggesting that fibroblasts may serve as precursor cells in ectopic bone formation^80^.

#### Changes in ECM mechanical characteristics trigger bone formation

Fibroblasts release matrix-associated components that modify the mechanical properties of ECM, contributing to ectopic bone formation.

Tenascin-C (TNC), a large extracellular matrix glycoprotein, is upregulated in ankylosing spondylitis (AS) and other inflammatory conditions^81^. Research indicates that TNC enhances Hippo/YAP signaling, reducing matrix adhesion while promoting chondrogenesis and pathological bone formation^82^. Li *et al.* reported that inflammation in AS leads to ECM remodeling, thereby accelerating pathological bone growth. Single-cell transcriptome analysis revealed that FSP1+ fibroblasts are the primary source of TNC in AS. When ligament-derived human fibroblasts were exposed to AS-associated inflammatory cytokines (TNF-α, IL-17A, IL-22), TNC expression increased at both mRNA and protein levels. TNC abnormalities disrupt ECM adhesion and mechanical signaling, inhibit YAP, and encourage endochondral ossification. Notably, administration of TNC-neutralizing antibodies in an AS mouse model significantly reduced pathological bone formation^82^.

Similarly, changes in ECM stiffness mediated by fibroblasts can contribute to HO. Calcified apoptotic vesicles (apoVs) are a heterogeneous group of membrane-bound vesicles involved in bone formation and mineral localization. These vesicles are released by apoptotic cells^83^. Gong *et al.* identified a specific subpopulation of fibroblasts, PROCR+ fibroblasts, which undergo apoptosis in the early stages of HO. These fibroblasts release calcified ApoVs into the ECM, where they accumulate and calcify^84^. Annexin channels help these apoVs enrich calcium, which adheres to collagen I via electrostatic interactions, forming calcified nodules that stiffen the ECM and lead to tendon calcification. This stiffening may activate the Hippo signaling pathway, promoting the differentiation of MSCs into osteogenic cells^85^. Moreover, the study demonstrated that culturing calcified ApoVs and macrophages on a stiff substrate induced macrophage polarization from M1 to M2. Inhibiting the release of calcified apoVs or reducing macrophage numbers delayed the onset and progression of HO^84^.

Calcium deposition mediated by fibroblasts has also been observed in neurogenic heterotopic ossification (NHO), a secondary injury that occurs following traumatic brain injury (TBI). NHO is characterized by greater severity and higher recurrence rates compared to general HO. Research indicates that pyroptosis is an important part of this process. Brain-derived extracellular vesicles (BEVs), released after TBI, can cross the blood-brain barrier and contribute to secondary injury in distant organs. Immunofluorescence staining revealed that BEVs, labeled with PKH26, were engulfed by fibroblasts labeled with phalloidin. Fibroblasts cultured with BEVs underwent pyroptosis, which promoted calcium deposition in tendons. Local microcalcification in injured tendons significantly increases extracellular matrix stiffness, which in turn triggers MSC osteogenic differentiation and promotes the M2 macrophage phenotype^86^.

### Fibroblast-mediated fibrosis provides a suitable microenvironment in HO

Following inflammation, fibroblasts accumulate in damaged tissue and play a critical role in tissue repair. These processes may result in impaired tissue repair and fibrosis^87^. In advanced fibrosis, progenitor cells may undergo abnormal differentiation, leading to HO.

#### Fibrosis in HO

HO has been observed in various fibrosis-related conditions, including keloids and abdominal midline scars^88^, ^89^. ICAM-1, an adhesion molecule expressed in fibroblasts, interacts with immune cells and can contribute to the development of dermatofibrotic diseases like scleroderma^41^. Studies suggest that scleroderma may increase the risk of HO. In one case, a patient with scleroderma exhibited significant calcification and ossification in the right papillary muscle and right masticatory muscle^57^.

A fibrotic state has also been observed in HO^77^. Vasconcellos *et al.* reported fibrosis in blast-injured tissues of both rats and humans, with elevated levels of fibronectin, SMAD3, PAI-1, and TGF-β1. Inflammatory cytokine markers, including ACTA, BMP1, BMP3, and TNF-α showed increased mRNA expression during fibrosis. Additionally, OPN, RUNX2, and COL1A1 levels were elevated in both rat and human tissues on days 7 and 10 after blast injury, suggesting that these fibrosis markers play a key role in tissue regeneration and abnormal osteogenesis^90^.

#### Hypoxia and fibrosis interact to amplify HO

Hypoxia-induced HIF-1α and VEGF signaling pathways are critical for chondrocyte survival, proliferation, and vascular development^91^. Additionally, TGF-β, PDGF, and IL1-β, which are involved in fibrosis and fibroblast activation, interact with the osteoblast-specific transcription factor Cbfa1/Runx2 in neonatal cartilage. This interaction promotes angiogenesis, thereby enhancing bone formation at abnormal sites^92^.

However, hypoxia and oxidative stress can also contribute to fibrosis and HO. Inhibitors of HIF hydroxylase have been shown to suppress the non-canonical TGF-β1 signaling pathway in the colon, reducing collagen synthesis and α-SMA levels in colonic fibroblasts^93^.

Idiopathic arthrofibrosis, which affects 3-4% of patients undergoing total knee arthroplasty (TKA), is characterized by excessive fibroblast proliferation and abnormal bone formation in the affected areas^94^, ^95^.

Freeman *et al.* identified three distinct areas within arthrofibrotic tissue. Two of these regions exhibited fibrosis, while the third, adjacent to the fibrotic areas, contained avascular fibrocartilage capable of forming bone via endochondral ossification^96^. Despite the oxygen deprivation conditions, apoptosis was absent, indicating that oxidative stress and fibroproliferative factors promote cell survival and proliferation^96^. Inflammatory oxidative stress is likely a contributing factor to the accumulation of mast cells, which release fibroblast growth factor (FGF) and chymotrypsin, stimulating fibroblast proliferation and generating hypoxic, avascular regions. Hypoxia and oxidative stress then drive the conversion of fibrous tissue into fibrocartilage, which generates heterotopic bone through endochondral ossification^96^.

In summary, the interplay between hypoxia and fibrosis seems to be a significant contributor to the progress of heterotopic bone formation.

### Fibroblasts serve as osteogenic precursor cells in HO

The FAP subpopulation comprises pluripotent cells that are PDGFRα+ and SCA1+, commonly found in muscle tissue. These cells are recognized for their capacity to produce fibrous tissue and adipocytes^97^. Several studies have shown that progenitor cells responsible for ectopic ossification in muscle often exhibit PDGFRα+ expression, in contrast to tendon cells. Wosczyna *et al.* reported that Tie2-Cre-labeled FAP contributed to 50% of ectopic bone and cartilage formation in mice^98^. Similarly, Eisner *et al.* showed that tissue-resident FAPs in skeletal muscle were the main source of osteoblasts in the BMP2-Matrigel mouse model of post-traumatic HO^99^.

#### Fibroblasts in AS

In AS, osteoblasts derived from MSCs are considered key contributors to abnormal bone formation. However, fibroblasts, as well as cells within ligaments and blood vessels, have also been identified as a possible source of ectopic bone formation.

Several inflammatory cytokines play a role in promoting the differentiation of AS-associated fibroblasts into osteogenic cells, leading to HO.

*In vitro* studies showed that treatment with recombinant human IFN-γ markedly elevated the mRNA and protein levels of MYC, ALP, and BMP2 in fibroblasts. Conversely, MYC knockdown reduced ALP and BMP2 expression. It was found that IFN-γ enhances MYC expression in fibroblasts within an inflammatory environment, subsequently binding to the promoters of ALP and BMP2 to upregulate their levels, converting fibroblasts into osteogenic cells^100^. In another study, BMP-2 binding to TGF-β1 increased TβRIII expression in AS fibroblasts. This sequentially activated the TGF-β1-Smad2/3 and BMP2-Smad1-RUNX2 signaling pathways, promoting the transformation of fibroblasts into osteoblasts^101^. Moreover, the inflammatory cytokine IL-6 upregulated annexin A2 expression in AS fibroblasts, activating the ERK signaling pathway and further stimulating osteogenic differentiation^102^.

miRNAs regulate mRNA activity and perform a critical function in the differentiation of fibroblasts into osteogenic cells, thereby contributing to abnormal bone formation. They achieve this by interacting with genes associated with AS.

Xiong *et al.* showed that miR-17-5p levels were markedly elevated in the fibroblasts and ligament tissues of AS patients comparing non-AS individuals. Their research demonstrated that miR-17-5p enhances the differentiation of fibroblasts into bone-forming cells by downregulating the 3' untranslated region (3'UTR) of the ANKH gene. They further proposed that miR-17-5p modulates cytokines such as Dickkopf-1 and VEGF, which may decelerate the progression of AS^103^. In another study, miRNAs were shown to inhibit the differentiation of fibroblasts into osteoblasts. Specifically, miR-214-3p targets the BMP2 gene, which activates the BMP-TGF-β signaling pathway and promotes osteogenic differentiation in AS fibroblasts by increasing the levels of MPR2, Smad5, p-Smad5, and OCN. Conversely, miR-214-3p directly targets BMP2 to suppress the osteogenesis of AS fibroblasts^104^.

#### Fibroblasts in OPLL

In OPLL, fibroblasts also exhibit the potential to form heterotopic bone. Fibroblasts from OPLL patients display osteoblast-like characteristics. Compared to non-OPLL fibroblasts, PERK (a single-pass type I endoplasmic reticulum membrane protein kinase, which is a major transducer of the endoplasmic reticulum stress response) was significantly upregulated in OPLL fibroblasts. Upon transfection with PERK-specific siRNA, mRNA expression of osteogenic markers such as OCN, ALP, and COLI was markedly reduced, indicating that PERK-mediated endoplasmic reticulum (ER) stress plays a critical role in the process of bone formation and regeneration of fibroblasts^105^.

Autophagy plays a key role in maintaining stem-like properties and regulating cell differentiation. The research indicated that fibroblasts derived from the ligaments of patients with OPLL exhibited elevated levels of autophagy in comparison to those from patients without OPLL. Beclin1-induced autophagy promotes osteogenic differentiation in ligament fibroblasts, which subsequently contributes to the advancement of OPLL^106^.

Similarly, leptin was found to stimulate autophagic activity in fibroblasts during osteogenic induction, enhancing osteogenesis, an effect that could be reversed by autophagy inhibition. Given the significance of the AMPK-BECN1 pathway in the osteogenic differentiation of stem cells, researchers introduced an AMPK inhibitor into the osteogenesis induction medium, leading to a marked reduction in mineralized nodule formation by fibroblasts. Additionally, Ocn, Col-1a1, and Runx2 expression levels were decreased in BECN1 knockdown cells compared to controls, and fibroblast mineralization was notably decreased following BECN1 knockdown. These findings show that activation of the AMPK/BECN1 pathway may contribute to OPLL progression by promoting autophagy in ligament fibroblasts^107^.

#### Fibroblasts in FOP

Fibroblasts have been identified as contributors to bone formation in FOP. Dimitra Micha *et al.* demonstrated the enhanced osteogenic potential of FOP fibroblasts using platelet lysate-based osteogenic transdifferentiation. This was evidenced by elevated expression of Sp7/Osterix, Runx2, ALP, and OC mRNA in primary dermal fibroblasts of FOP patients^108^. Following BMP4 stimulation in primary cultured fibroblasts from FOP patients and controls, SMAD1/5/9 phosphorylation occurred similarly in both groups. However, in FOP fibroblasts, but not in control fibroblasts, Activin A also induced SMAD1/5/9 phosphorylation. This suggests that FOP fibroblasts carrying mutated ACVR1 can respond to Activin A, triggering osteogenesis^109^.

#### Fibroblasts in a new form of HO

Researchers have recently discovered a new form of HO associated with a specific alteration in chromosome structure.

Enhancer hijacking typically occurs when changes in the chromosomal spatial structure reposition enhancers, allowing them to interact with trans-acting elements and significantly upregulate the expression of nearby genes^110^.

Melo *et al.* identified an inter-chromosomal insertional duplication in a female proband that disrupted a topologically associating domain (TAD), leading to a rare, aggressive form of HO characterized by extensive connective tissue ossification and premature death. The duplication event disrupts the TAD structure of the X chromosome, leading to a phenotype characterized by enhancer hijacking and overexpressed ARHGAP36 in fibroblasts. This overexpression of ARHGAP36 subsequently inhibits TGF-β signaling, activates hedgehog signaling, and upregulates the expression of genes and proteins involved in extracellular matrix production. Consequently, this cascade of events promotes the transformation of connective tissue into bone^111^.

## Fibroblast-associated molecules and signaling pathways in HO

### BMP/TGF-β signaling pathway

Bone morphogenetic protein (BMP), a member of the transforming growth factor-beta (TGF-β) superfamily, is well-recognized for its role in promoting bone formation. The BMP signaling pathway, extensively studied, is closely associated with the development of HO^21^, ^112^. TGF-β exerts diverse effects on bone formation, displaying both stimulatory and inhibitory actions on osteoblasts. Specifically, TGF-β stimulation induces excessive extracellular matrix production in osteoblasts and influences the migration of osteoblast-like cells toward the matrix, as well as the recognition of target cells^108^, ^113^. Additionally, once activated, TGF-β can modulate various cell types, including fibroblasts and immune cells^109^.

#### BMP and TGF-β are crucial in FOP

BMP and TGF-β stimulate fibroblasts to produce Activin A, a key factor in triggering FOP. The TGF-β superfamily plays a critical role in the signaling pathway in HO. In the mouse model of FOP, inhibiting TGF-β reduces HO^114^.

Ruiter *et al.* observed that TGF-β1 significantly upregulates INHBA expression and Activin A production in primary dermal fibroblasts from FOP patients. While BMP4 had a limited effect on Activin A production in normal fibroblasts and TGF-β1 had no effect, both TGF-β1 and BMP4 increased Activin A levels in FOP fibroblasts. Additionally, BMP4 has been shown to enhance TGF-β1 production in FOP fibroblasts. These findings indicate that TGF-β, a key co-regulator of Activin A, may contribute to FOP progression^109^.

#### BMP and TGF-β in fibrosis

BMP and TGF-β may contribute to the fibrotic microenvironment generated by fibroblasts in HO. As previously noted, Vasconcellos *et al.* identified elevated fibrosis markers in HO^90^. Fibroblast and myofibroblast activity, characterized by the deposition of collagen I, collagen III, and fibronectin, is a hallmark of nearly all forms of fibrosis^115^, ^116^.

Following trauma, BMP4 expression significantly increases in muscle tissue, enhancing MMP-1 protein levels. MMP-1, produced by fibroblasts, plays a critical role in extracellular matrix remodeling during damage repair and fibrosis^117^. Additionally, BMP4 modulates gene expression in mesenchymal pluripotent stem cells (MPCs), leading to upregulation of cartilage oligomeric matrix protein (COMP), an extracellular matrix-binding protein linked to tissue fibrosis^118^. Suggesting that BMP4 promotes fibrosis in response to muscle injury. Moreover, research indicates that the TGF-β/Smad signaling pathway is critical in stimulating collagen secretion from fibroblasts, further driving fibrosis development^116^.

Thus, activation of the BMP/TGF-β signaling pathway following trauma may stimulate fibroblasts to secrete collagen and other matrix components, aiding tissue repair. When this process is dysregulated, a fibrotic environment may develop, potentially facilitating heterotopic bone formation.

#### BMP and TGF-β in bone formation

BMP and TGF-β are key molecules that drive the osteogenic differentiation of fibroblasts, leading to HO. Targeting BMP2 and inhibiting the BMP-TGF-β axis, miR-2143p has been shown to prevent osteogenic differentiation in fibroblasts from patients with AS thereby inhibiting ectopic bone formation^104^. Similarly, suppression of miR-92b-3p reduces cell proliferation and bone formation in AS fibroblasts by inhibiting the BMP/Smad signaling pathway through increased level of transducer of erbB2 1 (TOB1)^119^.

To explore the role of Bmp2 in vascular and hematopoietic lineages, Prados *et al.* developed a transgenic mouse model where ectopic expression of Bmp2 is driven by the Tie2 promoter. These Tie2CRE/+; Bmp2tg/tg mice exhibited extensive soft tissue ossification, mirroring acquired HO, and genetic bone disorders like FOP^120^. Moreover, they found that hyperactive BMP2 signaling was linked to fibroblast accumulation and expansion of the Tie2+ fibro-adipogenic precursor cell population, suggesting that dysregulated BMP signaling in fibroblasts may play a pivotal role in ectopic bone formation^120^.

### Wnt signaling pathway

The classical Wnt signaling pathway is vitally important in abnormal bone growth. Wnt/β-catenin is generally regarded as a modulator of Hedgehog or BMP signaling^112^. BMP reduces Wnt signaling in osteoblasts by upregulating DKK1 and Sost through the MAPK and SMAD pathways, respectively. Moreover, the BMP and Wnt signaling pathways work in concert to regulate the osteogenic development of MSCs. Multiple studies have highlighted the key role of Wnt/β-catenin in promoting cell growth and differentiation of bone cells from fibroblasts.

In AS, CXCR4 enhances fibroblast proliferation and bone formation by activating the Wnt/β-catenin pathway^121^. In contrast, Dickkopf-1 (DKK1) inhibits this pathway in fibroblasts from AS patients, limiting both fibroblast proliferation and osteogenic potential^122^. On the other hand, miR-148a-3p promotes bone formation in AS fibroblasts by inhibiting DKK1 and activating Wnt signaling^123^. Additionally, AS patients exhibit downregulated expression of the ANKH regulator in their ligaments, while overexpression of ANKH has been shown to inhibit fibroblast mineralization and ossification. This effect is attributed to ANKH's regulation of ossification markers and its involvement in mediating the Wnt/β-catenin pathway^124^.

The interplay between the Wnt signaling pathway and other signaling pathways and other signaling cascades also promotes fibroblast proliferation and osteogenesis. For instance, the Wnt pathway interacts with PGE-2 signaling. Canonical Wnt proteins activate the PI3K/Akt pathway, resulting in the phosphorylation of GSK-3β and an increase in β-catenin levels. Similarly, PGE-2, through EP2 receptor activation, stimulates heterotrimeric G proteins, with the Gβγ subunit activating PI3-kinase and Akt. Akt then phosphorylates GSK-3β, preventing the degradation of β-catenin. Celastrol has been shown to inhibit PGE-2-induced osteogenic differentiation in AS fibroblasts and reduce fibroblast proliferation in a time- and dose-dependent manner^125^.

However, some studies suggest that Wnt pathway activation may inhibit ectopic bone formation. Chen *et al.* found that miR-497-5p in bone marrow mesenchymal stem cell (BMSC)-derived exosomes activates the Wnt/β-catenin pathway by targeting RSPO2, thereby accelerating the development of ossification of the OPLL^126^.

Furthermore, the Wnt signaling pathway is implicated in fibroblast-associated fibrosis^127^, though its role in injury-induced heterotopic ossification warrants further exploration.

### FGF signaling pathway

Fibroblast growth factor (FGF) binds to its receptor (FGFR) to regulate various cellular processes, including bone formation, and may influence the development of HO^128^.

Zhang *et al.*, through single-cell RNA sequencing, identified the FGF2 ligand-receptor pair, suggesting that FGF signaling pathways are implicated in the progression of heterotopic ossification of the elbow (HOE)^6^. However, FGF2 appears to have dose-dependent effects on ectopic bone formation. Yukio *et al.* inserted type I collagen disks containing a fixed amount of BMP-2 (5 µg) and varying concentrations of FGF-2 onto the back muscles of adult male mice. Their findings showed that low doses of FGF-2 facilitated BMP-2-induced ectopic bone formation by modulating the expression of bone morphogenetic protein receptors (BMPRs) on bone-forming progenitor cells, whereas high doses of FGF-2 inhibited bone formation^129^.

The role of FGF signaling in fibrosis is similarly complex, involving multiple FGF family members and receptors. Fibroblasts, as key mediators of fibrosis, can either promote or inhibit fibrotic development, impacting the progression of heterotopic ossification through the activation or inhibition of FGF signaling.

While the significance of FGF in HO is well established, further research is needed to clarify its dual role—whether promoting or inhibiting heterotopic bone formation and fibrosis—and to determine whether FGF signaling activation occurs within fibroblasts during this process.

#### FGF promotes ectopic bone formation

Freeman *et al.* demonstrated that mast cells secrete FGF, which stimulates fibroblast proliferation and chondrocyte differentiation in regions of arthrofibrosis following knee replacement surgery. This process contributes to the progress of fibrosis and the transformation of fibrous tissue into fibrocartilage, ultimately facilitating heterotopic bone formation through endochondral ossification^96^. Moreover, Zhang *et al.* found that Klotho reduced the levels of FGFR1 and phosphorylated extracellular signal-regulated kinase 1/2, delaying bone mineralization.

Fibrosis is a key contributor to ectopic bone formation, and FGF-mediated fibrosis has been observed across multiple organs. For instance, in a CCL4-induced liver fibrosis model, FGF15 knockout mice exhibited reduced liver fibrosis, suggesting that FGF15 plays a role in fibrotic progression^130^. In chronic kidney disease, elevated circulating levels of FGF23 are associated with pathological changes such as fibrosis, inflammation, and myocardial hypertrophy. FGF23 may directly act on cardiac fibroblasts via the FGFR4/PLCγ/calcineurin/NFAT signaling pathway, inducing cardiac hypertrophy^131^.

#### FGF inhibits ectopic bone formation

Sakano *et al.* implanted bone matrix powder (MP) into the right hamstring tendon of mice and administered varying doses (1, 10, 100 ng, and 1, 10 μg) of basic fibroblast growth factor (bFGF). The findings indicated that a 1 μg dose of bFGF significantly reduced the size of heterotopic bone, while a 10 μg dose completely inhibited ossification^132^. However, contrasting case reports have documented ectopic ossification in two patients treated with bFGF for sacral pressure ulcers. Upon discontinuation of bFGF and removal of the ossified tissue, the ulcers gradually shrank and eventually closed^133^.

FGF-2 also promotes the differentiation and specialization of MSCs derived from human periodontal ligament stem cells (hPDLSCs). Its primary role is to facilitate the specialization of hPDLSCs into tendon/ligament cells and counteract BMP-2 and BMP-4-induced hard tissue formation^134^. Furthermore, hyperphosphatemic familial tumoral calcinosis (HFTC), a rare and debilitating disease, results from impaired FGF23-mediated phosphate regulation, causing FGF23 deficiency or resistance, resulting in hyperphosphatemia and heterotopic calcifications^135^.

## The possible interaction between fibroblast and macrophage in HO

The degree of interaction between macrophages and fibroblasts—both essential for the development of HO—and the specific mechanisms through which they communicate to promote HO remain critical yet unresolved questions that warrant further investigation.

### In FOP

Several researches have highlighted the crucial role of macrophages role in FOP^136^. Barrue *et al.* observed that activation of mutant ACVR1 in FOP macrophages enhances NF-kB and p38 MAPK activity, contributing to inflammation, with the ACVR1 in FOP macrophages enhancing NF-κB and p38 MAPK activity, contributing to inflammation, with the mutant ACVR1 potentially acting in a TLR4-selective manner^57^. Another study found that this activation increases the production of TGF-β and pro-inflammatory cytokines, likely due to the upregulation of NF-κB and p38 MAPK pathways^136^. Elevated TGF-β levels subsequently attract MSCs to sites of HO, fostering the development of abnormal bone tissue.

FOP is particularly notable in that even minor inflammation can trigger significant HO, raising the question of whether a specific disease amplification loop is involved. We hypothesize that communication between fibroblasts and macrophages may contribute to the progressive worsening of FOP. Further investigation into this interaction could provide valuable insights into the disease mechanism.

**Figure 4:**
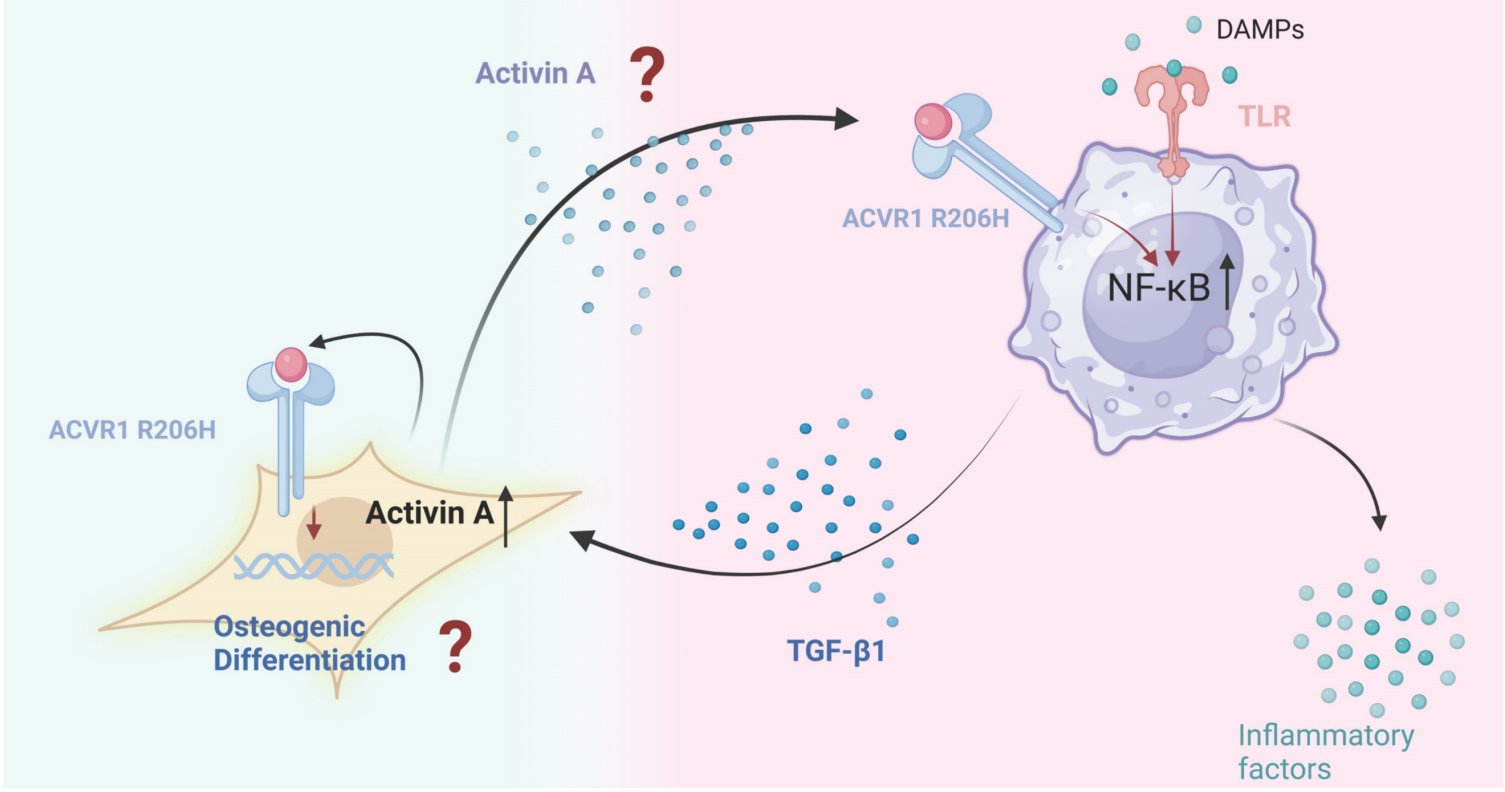
**The possible interaction between fibroblast and macrophage in FOP.** In FOP, injury triggers the release of DAMPs, leading to an intense inflammatory response. Macrophages with mutant ACVR1 and TLR4 receptors respond by activating the NF-κB signaling pathway, perpetuating an inflammatory state. This aberrant ACVR1 activation also elevates TGF-β and pro-inflammatory cytokine secretion. TGF-β1 acts as an agonist, driving Activin A production in fibroblasts, which further activates mutant ACVR1 receptors on macrophages. This sustains the inflammatory response and enhances TGF-β production. TGF-β, in turn, recruits MSCs and fibroblasts, promoting their osteogenic differentiation and contributing to ectopic bone formation. This process establishes a feedback loop, wherein fibroblasts produce more Activin A, maintaining chronic inflammation and continuous ectopic bone development. (Created in BioRender.com).

Following injury, FOP patients may exhibit a heightened inflammatory response due to the release of PAMPs or DAMPs. Mutant ACVR1 and TLR4 receptors on macrophages detect these signals, activating the NF-κB pathway through the regulation of TGF-β-activated kinase 4 (TAK1) expression, thereby triggering inflammation and promoting the release of TGF-β and pro-inflammatory cytokines^57^. TGF-β1 induces Activin A production in fibroblasts, which in turn stimulates mutant ACVR1 in macrophages, perpetuating the pro-inflammatory state and further enhancing TGF-β production^109^. TGF-β plays a pivotal role in promoting aberrant bone formation by recruiting MSCs and fibroblasts, driving their osteoblastic differentiation. Additionally, TGF-β contributes to a positive feedback loop that stimulates fibroblasts to produce more Activin A, sustaining chronic inflammation and facilitating ongoing ectopic bone formation **(Figure 4)**.

Several important questions remain unresolved. Specifically, it is unclear whether the mutant ACVR1 in macrophages can directly activate and modulate downstream inflammatory signaling pathways via Activin A. Furthermore, it is necessary to investigate whether Activin A, as a key mediator of fibrosis, extends the functional role of fibroblasts in the pathogenesis of FOP.

### In traumatic HO

In response to skeletal muscle trauma, hematomas form rapidly between the injured muscle fibers, and inflammatory cells, particularly macrophages, infiltrate the site. Macrophage development, differentiation, and proliferation in homeostasis depend on colony-stimulating factor 1 (CSF1), which is predominantly produced by fibroblasts. During fibrosis and inflammatory responses, fibroblasts maintain persistent CSF1 expression, ensuring the presence of resident or newly recruited macrophages^137^, ^138^ At the site of inflammation, macrophages release cytokines and growth factors, such as platelet-derived growth factor (PDGF), which interacts with PDGFRα on fibroblasts, promoting their proliferation^139^-^143^.

If the crosstalk between fibroblasts and macrophages is not properly resolved during wound healing, it may perpetuate inflammation, converting an acute response into chronic inflammation. This unresolved state can foster a fibrotic environment, potentially leading to abnormal bone formation as a result of impaired tissue repair. CCL2 is highly expressed in conditions characterized by monocyte infiltration, such as burn injuries—a major precursor for HO^144^, ^145^. CCL2 plays a pivotal role in early inflammation by recruiting and polarizing macrophages to the injury site. Early in the inflammatory process, fibroblasts release CCL2, attracting macrophages, which then secrete additional pro-inflammatory factors, including CCL2 and TGF-β. This triggers the TGF-β/Smad signaling pathway in fibroblasts, promoting collagen deposition and fibrosis. Furthermore, this signaling cascade influences osteoprogenitor cells, contributing to abnormal bone formation in affected areas.

**Figure 5:**
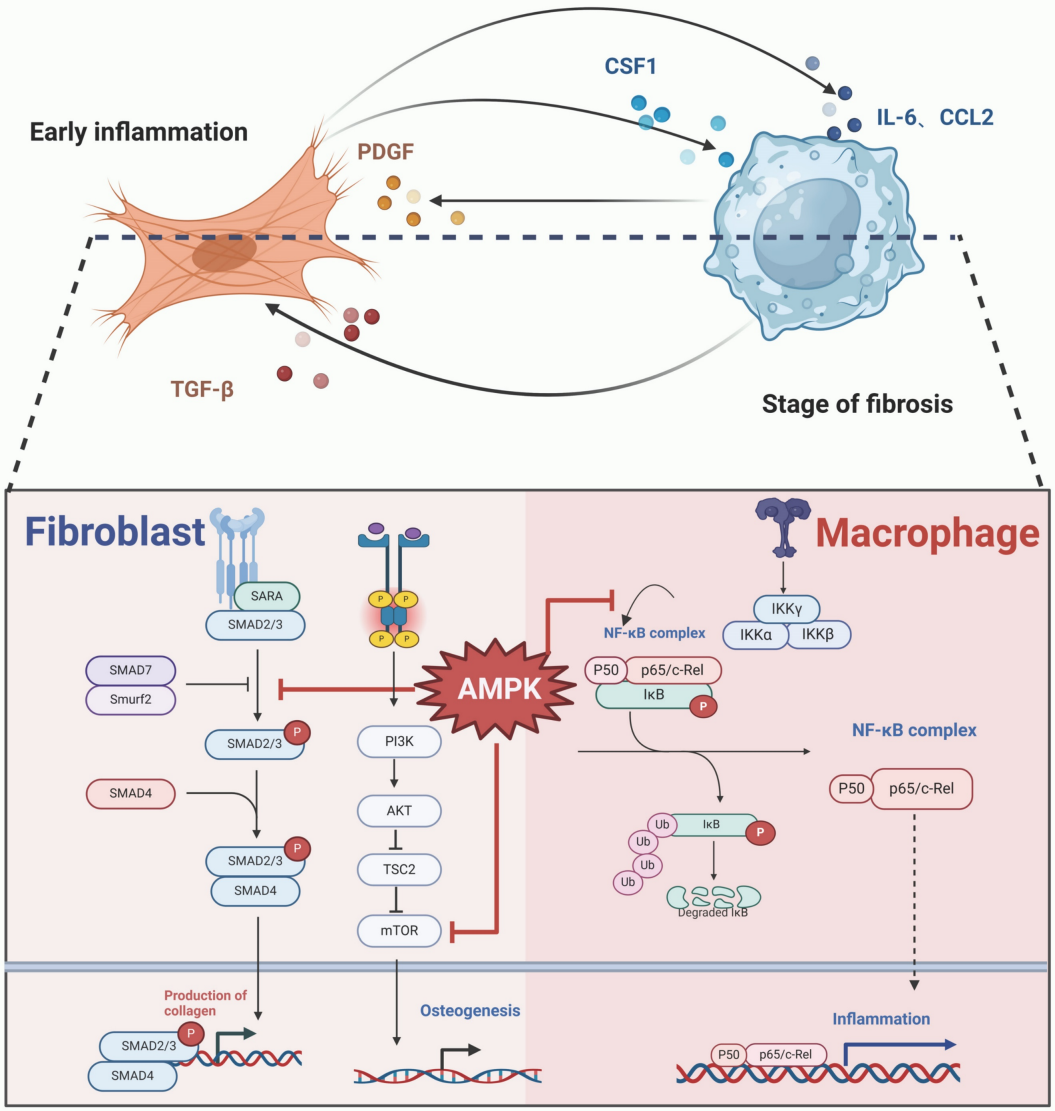
**AMPK signaling pathway in macrophages and fibroblasts cross-talk.** Activation of AMPK inhibits macrophage recruitment and activity, the progression of the inflammatory state via NF-κB signaling pathway inhibition, the fibrotic processes through Smad signaling suppression, and directly curtails osteogenesis of precursor cells by obstructing the mTOR signaling. (Created in BioRender.com)

The activation of the AMPK signaling pathway may play a pivotal role in preventing interactions between fibroblasts and macrophages. Research indicates that AMPK activation can decrease the level of phosphorylated NF-κB p65, resulting in reduced expression of IL-1β and CCL2 in macrophages^146^. Additionally, the anti-inflammatory effects of AMPK, which include the inhibition of the NF-κB signaling pathway, can be reversed by the NF-κB inhibitor QNZ^146^. In fibroblasts, AMPK activation inhibits the mTOR signaling pathway and decreases the levels of AKT, mMP2, and mMP9, consequently suppressing the migration of NIH 3T3 cells, a specific fibroblast line^147^. Furthermore, AMPK activation downregulates the TGF-β1/Smad3 pathway, leading to a reduction in type I collagen production in fibroblasts^147^. The activation of the AMPK/mTOR signaling pathway has also been proposed as an effective strategy to inhibit osteogenic differentiation in ectopic bone progenitor cells^148^. In the case of AS, the use of the AMPK activator metformin effectively reduced the osteogenic differentiation of fibroblasts and reduced the formation of ectopic bone^68^. **(Figure 5)**.

As previously mentioned, ApoVs secreted by PROCR+ fibroblasts in the early stages of HO contributes to tendon ECM calcification and stiffening. Notably, this stiffening of the Achilles tendon facilitates the polarization of M2 macrophages, which then triggers the osteogenic cascade during HO progression. Both inhibiting apoV release and depleting macrophages have been shown to successfully reverse HO^84^.

In summary, fibroblast-mediated alterations to the ECM not only promote the osteogenic differentiation of MSCs but also induce M2 macrophage polarization. M2 macrophage polarization is central to ectopic bone formation and influences MSCs or osteogenic precursor cells. The increased presence of Nestin+ MSCs in NHO mice, as observed in Lu *et al.*'s study, may result from M2 macrophages recruiting circulating MSCs by secreting IGF1and BMPs, ultimately leading to the formation of fully mature cancellous bone containing marrow^86^.

Further clarification is needed to determine whether fibroblasts, as cells with osteogenic differentiation potential, can be activated for osteogenesis in the M2 macrophage-polarized environment. The interactions between fibroblasts and macrophages—or, more broadly, between immune cells and osteogenic precursor cells—remain a central focus of our study.

## The main strategies for targeting fibroblasts in HO treatment

Currently, clinical interventions for HO are limited. Conventional surgical resection often leads to recurrence or exacerbation of the condition. Consequently, most cases are managed conservatively with steroids or NSAIDs, although the effectiveness of this approach is constrained, and the optimal timing for intervention is yet to be established. Therefore, identifying new therapeutic targets is essential^38^, ^39^.

Here, we discuss potential treatments aimed at modulating the role of fibroblasts at various stages of HO **(Table 1)**.

### Target fibroblast aging

Rapamycin, a macrolide molecule derived naturally, possesses immunosuppressive and anti-cancer properties^149^. At the molecular level, rapamycin forms a complex with the cytoplasmic protein FK506-binding protein 12 (FKBP12), inhibiting mTORC1 activity. This blocks the catalytic region of mTOR, preventing the phosphorylation of critical substrates such as ribosomal protein S6 kinase (S6K)^150^. Rapamycin has demonstrated efficacy in reducing HO by inhibiting the mTOR signaling pathway^151^-^153^.

As noted earlier, senescent fibroblasts in HO release SASPs through the PI3K/Akt/mTOR pathway. These SASPs influence surrounding cells, inducing their reprogramming into osteogenic progenitor cells, which contribute to HO development. Rapamycin effectively reduced SASP-related factors in senescent fibroblasts by 35%, leading to a reduction in heterotopic bone formation^4^.

Metformin, a widely used treatment of type 2 diabetes mellitus (T2D), is recognized for its potent activation of the AMPK signaling pathway and its broader effects, including cardiovascular benefits, lipid metabolism regulation, anti-aging properties, and HO prevention^148^, ^154^, ^155^.

Metformin can reduce cellular senescence and SASP release^156^. Petrocelli *et al.* reported that metformin mitigated the effects of cellular senescence and SASPs, reduced muscle atrophy, and regulated ECM remodeling in elderly individuals who underwent bed rest followed by walking bed rest followed by walking^157^.

### Target inflammation

Uncontrolled inflammation impairs tissue repair and regeneration, potentially leading to HO. Therapeutic strategies targeting leukocytes and inflammatory cytokines have introduced significant advances in the treatment of inflammatory disease. However, while these interventions reduce disease activity, they rarely provide a complete cure, with only a small proportion of patients achieving remission^158^. More recently, increasing recognition of the fibroblastic stroma's role in producing inflammatory mediators has highlighted the potential of targeting the stroma in therapeutic approaches.

#### Preventing inflammatory fibroblast activation

Given that fibroblasts respond to activation signals, understanding and inhibiting these pathways may offer a promising approach to targeting fibroblast activity in HO.

Fibroblasts contribute to HO development by participating in inflammatory responses, such as through NF-κB signaling activation, which promotes heterotopic bone formation in conditions like OPLL. Metformin has been shown to reduce NF-κB signaling in macrophages and inhibit BMP signaling in osteoblast progenitor cells, thereby preventing trauma-induced HO^146^. However, further research needs to determine whether metformin can also prevent abnormal bone formation by inhibiting NF-κB signaling in fibroblasts.

Additionally, AMPK activation has been shown to effectively stimulate PI3k and Akt expression in fibroblasts in AS, thereby mitigating inflammatory progression^68^. Thus, metformin may reduce abnormal bone growth in AS by suppressing the inflammatory response and inhibiting fibroblast-mediated bone cell differentiation.

#### Targeting fibroblast-derived effector molecules

Fibroblasts, being a major source of cytokines such as IL-6, can produce significant quantities of inflammatory factors in comparison to leukocytes. To effectively target these inflammatory fibroblasts, one promising strategy involves inhibiting the release of the vital cytokines and chemokines they secrete. By doing so, it may be possible to modulate the inflammatory response and address the underlying conditions driven by these fibroblasts.

As previously discussed, IL-6, a major inflammatory mediator produced by fibroblasts in response to muscle injury, plays a role in activating macrophages and strengthening their interaction. Blocking IL-6 could potentially disrupt this inflammatory cascade, allowing the inflammatory state to return to normal.

#### Nanomedicine in fibroblasts

Recently, cell membrane-camouflaged nanoparticles have gained attention as a potential therapeutic strategy for treating inflammatory diseases^159^. These nanoparticles can mitigate inflammation by neutralizing microorganisms or inflammatory cytokines through various mechanisms^160^. Given the important role of resident fibroblasts in the early stages of inflammation, fibroblast membrane-camouflaged nanoparticles may hold therapeutic potential in treating inflammation^161^.

Sun *et al.* engineered TLR4-presenting fibroblast membrane-camouflaged nanoparticles (DPC@NPs) that mimic the membrane functions of natural fibroblasts and are designed for early inflammatory intervention^162^. DPCs were genetically engineered to express high levels of TLR4 antigens in response to LPS stimulation, and their membrane was fused with PLGA nanoparticles to stabilize the cell membrane and prevent collapse^162^. The interaction between DPC@NPs and E. coli LPS inhibited the levels of several cytokines by deactivating key intracellular signaling pathways associated with inflammation.

### Target ECM remodeling

Collagen deposition properties present new opportunities for the targeted diagnosis and treatment of HO.

Similar to normal articular cartilage, HO lesions are predominantly composed of type II collagen (Col2a1)^163^. In this context, Wang *et al.* developed a novel near-infrared (NIR) fluorescent probe - WL-808 for early HO diagnosis. This probe, consisting of the NIR cyanine dye IR-80827 and the WYRGRL peptide, efficiently penetrates the cartilage extracellular matrix and specifically binds to Col2a1^164^.

Based on the WL-808, they explored the therapeutic potential of ROS generated by Photodynamic therapy (PDT) using 808 nm irradiation to inhibit HO cartilage and bone formation, both *in vitro* and *in vivo* studies. The result demonstrated that WL-808 selectively targeted the type II collagen cartilaginous framework in HO, inducing cell apoptosis and promoting ECM degradation upon NIR stimulation, thereby inhibiting ectopic bone formation^165^.

### Target fibrosis

Numerous studies have demonstrated that rapamycin can inhibit fibroblast proliferation, metabolic activity, and collagen formation in fibrosis-related diseases such as laryngotracheal stenosis and liver cirrhosis^166^.

Post-traumatic muscle damage frequently results in muscle fibrosis, myofibrillar atrophy, and the formation of heterotopic bone^90^. Research indicates that the administration of BMP is directly associated with increased muscle size and reduced atrophy, making it an effective strategy for mitigating muscle fiber atrophy following trauma. However, local BMP treatment often leads to the formation of heterotopic bone, complicating muscle repair. Agarwal *et al.* observed that in mice with heightened T1 BMPR activity (caAcvr1fl/fl) or targeted BMP administration, myofiber damage and fibrosis occurred before heterotopic bone formation. Rapamycin was found to reduce fibrosis and mesenchymal stromal cell accumulation at the injury site and thereby prevented fibrotic scarring and the development of heterotopic bone. A potential therapeutic strategy might involve the combined use of rapamycin and BMP signaling modulation to reduce or eliminate fibrosis, prevent ectopic bone formation, and enhance muscle fiber size following injury^167^.

Fibrosis in muscle tissue is often a precursor to abnormal bone formation in unwanted locations. Wang *et al.* found that metformin can activate AMPK, which in turn inhibits the overexpression of TGF-β1 and TGF-β1-induced phosphorylation of Smad2/3. This pathway effectively reduces muscle contractures and fibrotic tissue development within muscle^168^.

### Target bone formation

As previously noted, fibroblasts in FOP display a notable capacity for bone formation, indicating their potential role as progenitor cells in ectopic bone development in FOP^169^. Neutralizing antibodies against Activin A have emerged as a therapeutic strategy for FOP. These antibodies prevent Activin A from binding to mutant ACVR1 on aberrant bone progenitor cells, thereby inhibiting Smad1/5/8 signaling and slowing disease progression^170^. The anti-Activin A antibody (REGN2477) is currently in phase 2 clinical trials to assess its safety, tolerability, and efficacy in adult FOP patients^170^. Additionally, compounds such as maleic acid, fendiline hydrochloride, and dorsomorphin have demonstrated the ability to inhibit FOP by suppressing ALK2 activation^171^-^173^.

Certain nucleotide analogs, including miRNAs, have also been shown to reduce abnormal bone formation^174^, ^175^. For example, miR-214-3p inhibits fibroblast-driven osteogenesis and abnormal bone growth in^104^. Similarly, inhibiting miR-92b-3p effectively reduced fibroblast proliferation and bone tissue formation in AS by blocking the BMP/SMAD signaling pathway through upregulation of TOB1^119^.

The Wnt signaling pathway plays a crucial role in fibroblast proliferation. Dickkopf-1, a Wnt pathway inhibitor, has demonstrated the ability to suppress fibroblast proliferation and osteogenesis in AS patients^122^. Moreover, celastrol has been found to inhibit the interaction between PGE-2 and the Wnt signaling pathway, thereby reducing osteogenic differentiation in AS fibroblasts. Celastrol also demonstrated a time- and dose-dependent reduction in the fibroblast^125^.

## Perspectives and Conclusions

In summary, during the pathological process of HO, fibroblasts not only provide an ECM scaffold for new bone formation but under certain conditions also exhibit osteogenic potential. Through interactions with other cells and factors, they regulate the initiation and progression of HO. Thus, fibroblasts are key targets in HO research, and modulating their activity and differentiation pathways may offer new strategies for preventing and treating HO. However, unresolved questions remain regarding the role of fibroblasts in HO.

Firstly, the lack of specific markers for fibroblasts poses a significant challenge. Although fibroblasts play critical roles in tissue repair, inflammation, and fibrosis, identifying specific markers for these cells remains difficult^176^. Fibroblasts are often indirectly identified by their morphology, functional responses under certain conditions, and expression of non-specific markers (e.g., vimentin, α-SMA, and collagen). These markers are also common in other cell types, particularly myofibroblasts, smooth muscle cells, and certain immune cells, complicating precise identification and functional studies. Additionally, fibroblasts are morphologically indistinguishable from MSCs, sharing similarities in immunophenotype, proliferation, differentiation potential, gene expression, and immunomodulatory capacities. Some researchers have even hypothesized that fibroblasts may be senescent MSCs, challenging traditional cell classifications^177^. Given the functional and phenotypic overlap, it is crucial to clarify whether fibroblast research encompasses MSCs.

In recent years, single-cell RNA sequencing data have shed light on differences in fibroblast surface marker expression. While no single marker can definitively distinguish all fibroblasts from other cell types, including MSCs, combinations of markers can offer broad distinctions. For instance, pan-fibroblast characteristics are defined by combinations such as PDGFRa, Dpt, and Pi16 or Col15a1^43^. A study using scRNA-seq to explore fibroblast heterogeneity in keloids identified a mesenchymal fibroblast subpopulation expressing markers like COL11A and POSTN, involved in skeletal development, ossification, or osteoblast differentiation. Additionally, in keloids, myofibroblasts were enriched within this mesenchymal fibroblast subpopulation. As previously mentioned, case reports have documented heterotopic ossification in keloid patients, suggesting that the increase in mesenchymal fibroblasts in keloids may indicate a link between skin fibrosis and bone or cartilage formation, aligning with these clinical findings^41^.

However, relying on scRNA-seq data to define fibroblast subpopulations presents challenges. For example, some identified "subtypes" may reflect developmental trajectory states or responses to stress or injury rather than distinct steady-state functions. Furthermore, most transcriptomic data are derived from mouse models, and fibroblast heterogeneity varies significantly across species. Therefore, whether many fibroblast subtypes identified in research are present in humans remains unclear. The role of different fibroblast subpopulations in various pathological processes of HO remains to be determined, and the uncertainty surrounding fibroblast heterogeneity in HO may pose challenges for targeted therapies.

Beyond the fibroblast heterogeneity, antifibrotic therapies, immune regulation, tissue repair, and aging discussed in this paper, there is a lack of extensive research on the metabolic reprogramming and epigenetic mechanisms of fibroblasts in HO. In fibrotic processes, fibroblasts exhibit metabolic reprogramming, particularly enhanced glycolysis. Metabolic regulation not only affects fibroblast proliferation but may also influence the secretion of cytokines, thus modulating the behavior of surrounding cells^178^. In terms of epigenetics, mechanisms such as DNA methylation and histone modification may impact fibroblast activation and senescence. Furthermore, epigenetic regulation influences fibroblast plasticity and transdifferentiation potential. For instance, in wound healing and fibrosis, fibroblasts exhibit transdifferentiation abilities to other cell types (e.g., myofibroblasts or cancer-associated fibroblasts) through changes in epigenetic modifications^179^. Thus, targeting epigenetic regulation could offer promising therapeutic potential for treating HO in the future.

## Funding

This work was supported by the National Nature Science Foundation of China (32460216 to HL), and, Nature Science Foundation of Jiangxi Province of China (20224ACB206024, 20232BAB206081 and 20232BCJ23008to HL).

## Figures and Tables

**Table 1:** Therapeutic strategies against fibroblast in HO clinic treatment

Therapeutic agent	Description	Outcomes	Citation
Rapamycin	mTOR inhibitor	Targeting fibroblast aging, fibrosis, and bone formation	151-153
Metformin	AMPK activator	Targeting fibroblast aging, inflammation fibrosis, and bone formation	146
DPC@NPs	Fibroblast membrane-camouflaged nanoparticles	Targeting inflammation	162
WL-808	NIR fluorescent probe	Targeting ECM remodeling, and HO diagnosis	165
REGN2477	Neutralizing antibody	Preventng Activin A from binding to mutant ACVR1 on aberrant bone progenitor cells	170
Maleic acid, fendiline hydrochloride and dorsomorphin	ALK2 inhibitor	Targeting bone formation	171-173
MiR-214-3p	miRNA	Targeting bone formation	104
Dickkopf-1	Wnt inhibitor	Targeting bone formation	122
Celastrol	Wnt inhibitor	Targeting bone formation	125

## Abbreviations

HO: Heterotopic ossification
NSAIDs: nonsteroidal anti-inflammatory drugs
ECM: extracellular matrix
POH: progressive osseous heteroplasia
AHO: albright hereditary osteodystrophy
FOP: fibrodysplasia ossificans progressive
PTH: parathyroid hormone
Gsα: G protein alpha subunit
BMP: bone morphogenetic protein
TGF-β: transforming growth factor-beta
MSCs: mesenchymal stem cells
EMT/EndMT: epithelial/endothelial-mesenchymal transformation
FRCs: fibroblastic reticular cells
CAFs: cancer-associated fibroblasts
SASP: senescence-associated secretory phenotype
FAP: the fibro/adipogenic progenitor
NF-κB: nuclear factor kappa-light-chain-enhancer of activated B cells
DAMPs: damage-associated molecular patterns
PAMPS: pathogenesis-associated molecular patterns
OPLL: ossification of the posterior longitudinal ligament
Cx43: connexin 43
AMPK: adenosine 5'-monophosphate activated protein kinase
AS: ankylosing spondylitis
DMP1: dentin matrix protein 1
SOST: sclerostin
MMP-9: matrix metallopeptidase
RECK: reversion-inducing cysteine-rich protein with kazal motifs
LF: ligamentum flavum
LSP1: lymphocyte-specific protein 1
BCP: Biphasic calcium phosphate
IGF1: insulin-like growth factor 1
TNC: tenascin- c
NHO: neurogenic heterotopic ossification
TBI: traumatic brain injury
BEV: brain-derived extracellular vesicles
TKA: total knee arthroplasty
FGF: fibroblast growth factor
3'UTR: 3' untranslated region
ER: endoplasmic reticulum
TAD: topologically associating domain
MPCs: mesenchymal pluripotent stem cells
COMP: cartilage oligomeric matrix protein
TOB1: transducer of erbB2 1
DKK1: dickkopf-1
BMSCs: bone marrow mesenchymal stem cell
HOE: heterotopic ossification of the elbow
BMPRs: bone morphogenetic protein receptors
MP: matrix powder
bFGF: basic fibroblast growth factor
hPDLSCs: human periodontal ligament stem cells
HFTC: hyperphosphatemic familial tumoral calcinosis
TAK4: TGF-β-activated kinase 4
CSF1: colony-stimulating factor 1
PDGF: platelet-derived growth factor
T2D: type 2 diabetes mellitus
FKBP12: FK506-binding protein 12
S6K: ribosomal protein s6 kinase
PLGA: poly(lactic glycolic acid)
PDT: photodynamic therapy
NIR: near-infrared
